# *Acidithiobacillus ferrooxidans *metabolism: from genome sequence to industrial applications

**DOI:** 10.1186/1471-2164-9-597

**Published:** 2008-12-11

**Authors:** Jorge Valdés, Inti Pedroso, Raquel Quatrini, Robert J Dodson, Herve Tettelin, Robert Blake, Jonathan A Eisen, David S Holmes

**Affiliations:** 1 Center for Bioinformatics and Genome Biology, Fundación Ciencia para la Vida and Depto. de Ciencias Biologicas, Facultad de Ciencias de la Salud, Universidad Andres Bello, Santiago Chile; 2 J. Craig Venter Institute, Rockville, MD, USA; 3 The Institute for Genomic Sciences, University of Maryland, Baltimore, MD, USA; 4 University of California Davis Genome Center, Section of Evolution and Ecology, U.C. Davis, Davis, CA, USA; 5 University of California Davis Genome Center, Dept of Medical Microbiology and Immunology, U.C. Davis, Davis, CA, USA; 6 Division of Basic Pharmaceutical Sciences, Xavier University, New Orleans, LA, USA

## Abstract

**Background:**

*Acidithiobacillus ferrooxidans *is a major participant in consortia of microorganisms used for the industrial recovery of copper (bioleaching or biomining). It is a chemolithoautrophic, γ-proteobacterium using energy from the oxidation of iron- and sulfur-containing minerals for growth. It thrives at extremely low pH (pH 1–2) and fixes both carbon and nitrogen from the atmosphere. It solubilizes copper and other metals from rocks and plays an important role in nutrient and metal biogeochemical cycling in acid environments. The lack of a well-developed system for genetic manipulation has prevented thorough exploration of its physiology. Also, confusion has been caused by prior metabolic models constructed based upon the examination of multiple, and sometimes distantly related, strains of the microorganism.

**Results:**

The genome of the type strain *A. ferrooxidans *ATCC 23270 was sequenced and annotated to identify general features and provide a framework for *in silico *metabolic reconstruction. Earlier models of iron and sulfur oxidation, biofilm formation, quorum sensing, inorganic ion uptake, and amino acid metabolism are confirmed and extended. Initial models are presented for central carbon metabolism, anaerobic metabolism (including sulfur reduction, hydrogen metabolism and nitrogen fixation), stress responses, DNA repair, and metal and toxic compound fluxes.

**Conclusion:**

Bioinformatics analysis provides a valuable platform for gene discovery and functional prediction that helps explain the activity of *A. ferrooxidans *in industrial bioleaching and its role as a primary producer in acidic environments. An analysis of the genome of the type strain provides a coherent view of its gene content and metabolic potential.

## Background

*Acidithiobacillus ferrooxidans *is a Gram-negative, γ-proteobacterium that thrives optimally at 30°C and pH 2, but can grow at pH 1 or lower [1]. It is abundant in natural environments associated with pyritic ore bodies, coal deposits, and their acidified drainages [2,3]. It is an important member of microbial consortia used to recover copper via a process known as bioleaching or biomining [4].

In a typical bioleaching operation, copper ore is first pulverized and placed in heaps. The heaps are then sprinkled with sulfuric acid and aerated to promote the microbial oxidation of iron and sulfur compounds. Some bioleaching heaps are very extensive; for example, the Escondida mine in northern Chile is putting into operation a heap that is 5 km long by 2 km wide and 126 m high (David Dew, personal communication). With a volume of a little more than one trillion (10^12^) liters, this bioleaching heap is arguably the world's largest industrial bioreactor.

**Figure 1:**
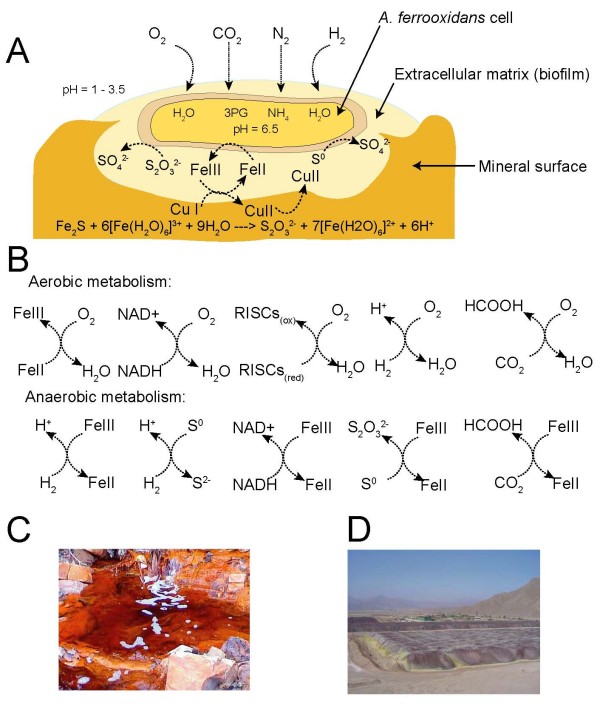
***A. ferrooxidans *and its proposed role in bioleaching**. The chemolithoautotrophic metabolism of *A. ferrooxidans *results in the oxidation/reduction of iron and sulfur compounds and the solubilization of copper and other commercially valuable metals in a process called bioleaching or biomining. It also results in the production of acidified solutions in pristine environments and acid mine drainage in bioleaching operations. A) Model of copper bioleaching by *A. ferrooxidans*. B) Oxidation/reduction reactions carried out by *A. ferrooxidans*. The scheme provided here presents the basic concepts of bioleaching and further details are provided in the review [4]. C) Acid mine drainage in the Rio Tinto, Spain, derived from naturally occurring pyritic ore bodies and abandoned mine workings initiated in pre-Roman times [3]. D) Commercial bioleaching heap for copper recovery, Chile. 3PG: 3-phosphoglycerate.

Bioleaching of copper ores is a two-step process: first, the biological oxidation of Fe(II) to produce Fe(III); second, the chemical oxidation of Cu(I) to the more soluble Cu(II) by Fe(III) which is reduced to Fe(II) in the process. *A. ferrooxidans *plays a key role by reoxidizing the Fe(II) to Fe(III), thus completing the cycle and allowing bioleaching to continue (Figure 1). The sulfuric acid produced by the biological oxidation of reduced sulfur compounds also promotes the solubilization of the Cu(II). Copper is recovered from this acidic solution using physico-chemical technologies such as solvent extraction and electroplating.

Bioleaching accounts for 10% of the copper production worldwide and is especially important as a technology for ores with a low percentage of copper that are otherwise uneconomical to extract. Another attractive feature of bioleaching is that it does not produce pollutants such as sulfur dioxide and arsenic that result from smelting. However, bioleaching does generate acid mine drainage that must be managed to prevent its release into the environment. The importance of bioleaching is likely to increase in the future as the mineral industry exploits ore deposits with lower copper content as richer ores become depleted. The increasing importance of bioleaching as a biotechnological process is stimulating increasing interest in the biology of *A. ferrooxidans *and associated bioleaching microorganisms.

*A. ferrooxidans *is one of the few microorganisms known to gain energy by the oxidation of ferrous iron in acidic environments, using the low pH of its natural environment to generate reverse electron flow from Fe(II) to NADH [5-8]. It can also obtain energy by the oxidation of reduced sulfur compounds, hydrogen, and formate [9,10]. The microorganism makes an important contribution to the biogeochemical cycling of metals in the environment and has the potential to assist in the remediation of metal contaminated sites by its ability to oxidize and reduce metals. Ferric iron and sulfuric acid are major by-products of its energy-transducing processes, and these chemicals can mobilize metals in the environment including toxic metals such as arsenic [11]. It can also reduce ferric ion and elemental sulfur, thus promoting the recycling of iron and sulfur compounds under anaerobic conditions [12,13]. Since the microorganism can also fix CO_2 _and nitrogen, it is thought to be a primary producer of carbon and nitrogen in acidic, nutrient-poor environments [14-17].

The study of *A. ferrooxidans *offers exceptional opportunities to probe life in extremely acidic environments. It may also offer insights into ancient ways of life in Archaean, euxinic, acidic seas [18] and suggest potential biomarkers to be used when searching for evidence of extra-terrestrial life [19]. One of its unusual properties is its ability to aerobically oxidize solid substrates such as pyrite (FeS_2_). Since the substrate cannot enter the cell, initial electron removal must take place either within the outer cell membrane or completely outside the cell. Although a substantial body of information exists regarding the use of solid minerals as electron sinks for biological processes (e.g., the reduction of ferric iron [20]), considerably less is known about how microorganisms recognize, attach to, and extract electrons from solid substrates. Investigations into the fundamental interactions between bacteria and mineral surfaces are critical for understanding the intricacies of interfacial biochemistry, biofilm formation, bacterial recognition of mineral surfaces, and the dispersal of microorganisms in the environment.

*A. ferrooxidans *thrives in mineral rich, acid environments where the concentration of dissolved ferrous iron can be as high as 10^-1 ^M, about 10^16 ^times that found in circum-neutral environments. The abundance of soluble iron has the potential to pose severe oxidative stress that could lead to DNA and protein damage via the Fenton reaction. This prompts questions as to the mechanisms that *A. ferrooxidans *employs for iron assimilation and homeostasis [21,22] and how it balances its use of iron as both a micronutrient and as a required energy source. In its natural environment, it must also confront unusually severe toxicity due to the high concentration of dissolved metals (e.g., copper, arsenic, mercury).

Although the internal pH of *A. ferrooxidans *is about pH 6.5, proteins that are either wholly or partially outside the inner membrane must function at pH 1–2, raising fundamental questions regarding how they fold and make protein-protein contact when confronted with such an extremely high proton concentration. It also raises questions as to how proton-driven membrane transport and energy processes function in the face of a proton motif force (pmf) across the inner membrane that is several orders of magnitude higher than typically found in neutrophilic environments.

Unfortunately, the lack of a well-developed system for genetic manipulation has prevented thorough exploration of the molecular biology and physiology of *A. ferrooxidans*. A bioinformatics-based analysis of its genome offers a powerful tool for investigating its metabolism. However, many of the earlier investigations of its genetics and metabolism were carried out on a variety of strains, some of which may be only distantly (or not at all) related to *A. ferrooxidans*. This allows the possibility that some experimental results, including enzyme identifications were not reliable indicators of the metabolism of the species. Genomic analysis of the type strain of *A. ferrooxidans *can provide a more coherent view of the gene content and metabolic potential of the species.

An analysis of amino acid metabolism based on the draft genome sequence of *A. ferrooxidans *ATCC 23270 was previously reported [23]. Here we present a complete, genome-based blueprint of the metabolic and regulatory capabilities of *A. ferrooxidans *and relate these findings to its unique lifestyle. This analysis will add to our understanding of the biochemical pathways that underpin the biogeochemical processes, metabolic functions, and evolution of microbial communities in acidic environments. This information also advances our understanding of the role of *A. ferrooxidans *in industrial bioleaching.

## Results and discussion

### 1. Genomic properties

**Figure 2:**
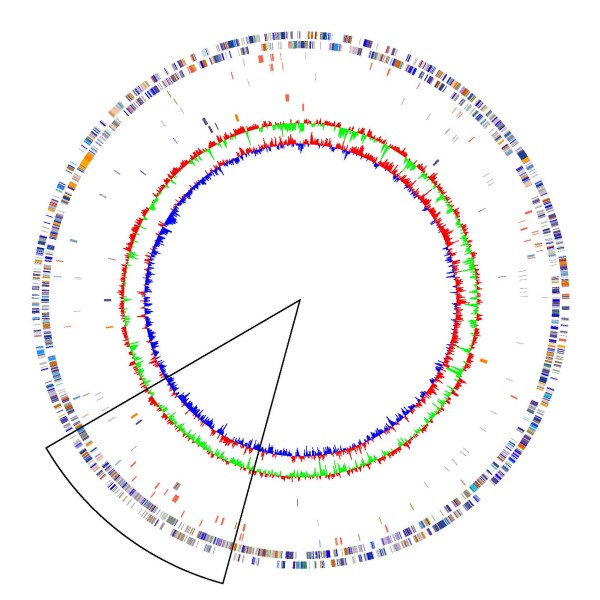
**Circular representation of the *A. ferrooxidans *ATCC 23270 genome sequence**. The two outer circles represent predicted protein encoding-genes on the forward and reverse strands, respectively. Functional categories are indicated by color, as follows: energy metabolism (green), DNA metabolism (red), protein synthesis (magenta), transcription (yellow), amino acid metabolism (orange), central intermediary metabolism (dark blue), cellular processes (light blue), nucleotide metabolism (turquoise), hypothetical and conserved hypothetical proteins (grey), mobile and extra-chromosomal elements (black), and general functions (brown). The third and fourth circles (forward and reverse strands) indicate major transposases and mobile elements (orange), plasmid-related genes (red), and phage elements (blue). The fifth and sixth circles (forward and reverse strands) indicate tRNA genes (gray). The seventh and eighth circles (forward and reverse strands) show genes predicted to be involved in sulfur (purple), iron (red), and hydrogen (orange) oxidation. The ninth and tenth circles show genomic GC bias and GC skew, respectively.

The genome of *A. ferrooxidans *ATCC 23270 (type strain) consists of a single circular chromosome of 2,982,397 bp with a G+C content of 58.77%. No plasmids were detected in the type strain, although they occur in several other strains of [24]. A total of 3217 protein-coding genes (CDSs) were predicted, of which 2070 (64.3%) were assigned a putative function (Table 1 and Figure 2). The genome encodes two ribosomal operons and 78 tRNA genes. A putative origin of replication (Figure 2) has been identified from marginal GC skew variations in the genome and by the localization of the *dnaN *and *dnaA *genes (AFE0001 and AFE3309).

**Table 1:** General features of the *A. ferrooxidans *ATCC 23270 genome.

**Characteristic**	**Value**
Complete genome size, bp	2,982,397
G+C percent (%)	58.77
Total number of CDSs	3,217
Coding density (%)	97.45
No. of rRNA operons (16S-23S-5S)	2
No. of tRNA genes	78
Proteins with known function	2,070
Conserved hypothetical proteins	388
Hypothetical proteins	759

**Most represented functional categories**	**(%)**

Cell envelope	7.8
Transport and binding proteins	7.61
Energy metabolism	6.52

**Best BLASTP comparisons against complete proteomes**	**Number of best blast hits**

γ-proteobacteria	899
β-proteobacteria	791
α-proteobacteria	271
δ-proteobacteria	103
Cyanobacteria	73
Archaea	41

### 2. Chemolithoautotrophy

*A. ferrooxidans *has a complete repertoire of genes required for a free-living, chemolithoautotrophic lifestyle, including those for CO_2 _fixation and nucleotide and cofactor biosynthesis (Additional file 1). Analysis of an earlier draft genome had predicted genes for the pathways for synthesis of most amino acids, although ten genes were missing [23]. Seven of these missing assignments have now been detected: a potential 6-phosphofructokinase in the glycolysis pathway (EC 2.7.1.11; AFE1807), pyruvate dehydrogenase (EC 1.2.4.1; AFE3068-70); shikimate kinase in the chorismate synthesis pathway and required for tryptophan, phenylalanine and tyrosine biosynthesis (EC 2.7.1.71; AFE0734); homeserine kinase in the threonine biosynthesis pathway (EC 2.7.1.39; AFE3097); N-acetyl-gamma-glutamil-1-phosphate reductase in the ornithine biosynthesis pathway and required for proline biosynthesis (EC 1.2.1.38; AFE3073); pirroline-5-carboxilate reductase involved in proline biosynthesis (EC 1.5.1.2; AFE0262); and asparagine synthase (EC 6.3.5.4: AFE1353). The three genes identified in *E. coli *which have not been found in *A. ferrooxidans *encode ornithine cyclodeaminase (EC 4.3.1.12) involved in proline biosynthesis, aromatic-amino-acid transaminase (EC 2.6.1.57), and arogenate dehydrogenase involved in tyrosine biosynthesis (EC 1.3.1.43).

*A. ferrooxidans *has two glutamyl-tRNA synthetases: a more discriminating one (D-GluRS, AFE0422) that charges only Glu-tRNA(Glu) and a less discriminating one (ND-GluRS, AFE2222) that charges Glu-tRNA(Glu) and Glu-tRNA(Gln). The latter one is a required intermediate in protein synthesis in many organisms [25]. An indirect regulation of glutamyl-tRNA synthetase by heme status suggests a potential metabolic connection between heme requirements, nitrogen, and central carbon metabolism [26].

Bioinformatic analysis supports prior experimental evidence that *A. ferrooxidans *has a versatile aerobic metabolism, capable of providing energy and reducing power requirements from inorganic compounds by the oxidation of Fe(II), reduced sulfur compounds, formate, and hydrogen. In addition, gene function predictions suggest that the microorganism is capable of anaerobic or micro-aerophilic growth using Fe(III) or elemental sulfur as alternative electron acceptors [27]. Many of the predictions were experimentally validated in a piece-meal fashion in a number of diverse strains of *A. ferrooxidans*, some of which may not belong to the same species [28]. Herein, we describe a coherent view of the metabolic potential of the type strain that will now allow a systematic appraisal of the diversity of the metabolic capacity of the *A. ferrooxidans *pangenome.

#### 2.1 CO_2 _fixation

*A. ferrooxidans *fixes CO_2 _via the Calvin-Benson-Bassham reductive pentose phosphate cycle (Calvin cycle) using energy and reducing power derived from the oxidation of iron or sulfur [29]. Early studies showed a relationship between the rate of iron and sulfur oxidation and the rate of CO_2 _fixation in *A. ferrooxidans *(no strain designated) [30]. Several enzymes of the Calvin cycle have been described in *A. ferrooxidans*, including the key D-ribulose-1,5-bisphosphate carboxylase/oxygenase (RuBisCO) [29]. Two structurally distinct forms of RuBisCO (I and II), with different catalytic properties, are typically present in autotrophs [31]. Genes encoding Form I (AFE3051-2) have been cloned and characterized from *A. ferrooxidans *[32,33]. Gene clusters potentially encoding a second copy of Form I (AFE1690-1) and a copy of Form II (AFE2155) were predicted and shown to be differentially expressed depending on whether *A. ferrooxidans *was grown on iron- or sulfur-containing medium. [34]. A gene predicted to encode a novel Rubisco-like protein known as Form IV [35] was recently identified in the genome (AFE0435) and is suggested to be involved in stress response (Esparza-Mantilla, personal communication) (Additional file [Supplementary-material S2]).

The genomic organization of the three gene clusters encoding the Rubisco type I and II enzymes in *A. ferrooxidans *is similar to that found in *Hydrogenovibrio marinus *strain MH-110, an obligate chemolithoautotrophic, hydrogen-oxidizing, marine bacterium. In *H. marinus*, these three-gene clusters are regulated in response to CO_2 _concentration, suggesting the ability to adapt to environmental conditions with different levels of CO_2 _[36].

#### 2.2 Energy metabolism

##### 2.2.1 Aerobic Iron oxidation

Since ferrous iron [Fe(II)] is rapidly oxidized by atmospheric oxygen at neutral pH, iron exists primarily in the oxidized form [Fe(III)] in aerobic environments. Therefore, ferrous iron is available for microbial oxidation principally in acidic environments where chemical oxidation is slow and Fe(II) is soluble, in anoxic conditions such as in marine sediments and at the interface between aerobic and anaerobic atmospheres [37]. In anoxic conditions, phototrophic bacteria can use light energy to couple the oxidation of Fe(II) to reductive CO_2 _fixation. Although little is known about the mechanisms involved, this process has been postulated to be an ancient form of metabolism and to represent a transition step in the evolution of oxygenic photosynthesis [38,39].

**Figure 3:**
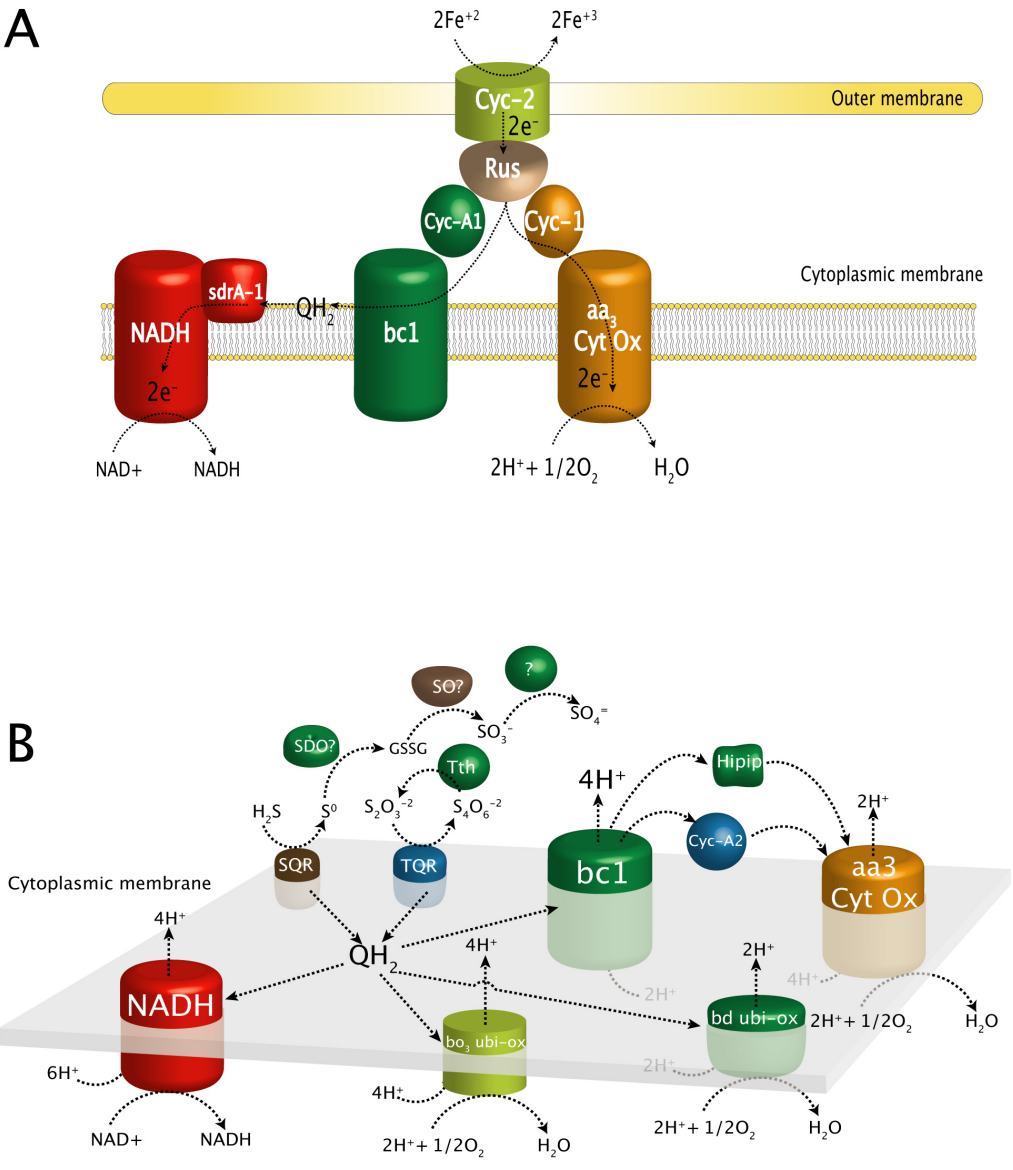
**Genome-based models for the oxidation of ferrous iron and reduced inorganic sulfur compounds (RISCs)**. Schematic representation of enzymes and electron transfer proteins involved in the oxidation of (A) ferrous iron and (B) reduced inorganic sulfur compounds (RISCs). Proteins and protein complexes are described in the text.

The bioinformatics analysis of the genome sequence of *A. ferrooxidans *has permitted the identification of the main components of the electron transport chain involved in iron and sulfur oxidation (Figure 3). Genes encoding iron oxidation functions are organized in two transcriptional units, the *petI *and *rus *operons. The *petI *operon (*petC-1, petB-1, petA-1, sdrA-1*, and *cycA-1; *AFE3107-11) encode the three subunits of the *bc*_1 _complex (PetCAB), a predicted short chain dehydrogenase (Sdr) of unknown function, and a cytochrome *c*_4 _that has been suggested to receive electrons from rusticyanin and pass them to the *bc*_1 _complex [5]. The *petI *operon has been analyzed experimentally in *A. ferrooxidans *strain ATCC 19859 [5] and recently in strain ATCC33020 [8].

The *rus *operon (*cyc2, cyc1, hyp, coxB, coxA coxC, coxD*, and *rus*; AFE3146-53) encodes two c-type cytochromes (Cyc1 and Cyc2), components of the aa3-type cytochrome oxidase (CoxBACD), and rusticyanin, respectively [40]. Cyc2 has been shown to accept electrons directly from Fe(II) and, given its location in the outer membrane, may carry out the first step in Fe(II) oxidation [41]. These proteins are thought to form a "respiratory supercomplex" that spans the outer and the inner membranes and transfers electrons from iron (or pyrite) to oxygen [40,42,43]. Based on transcriptional, biochemical, and genetic studies [28], it was proposed that electrons from ferrous iron oxidation flow through Cyc2 to rusticyanin. From there, some of the electrons feed the "downhill electron pathway" through *c*-cytochrome Cyc1 to *aa*_3 _cytochrome oxidase, some the "uphill electron pathway" that regenerates the universal electron donor NADH by the reverse electron flow through *c*-cytochrome CycA1--> *bc*_1 _complex-->ubiquinone pool-->NADH dehydrogenase (Figure 3a).

Genome analysis suggests a solution to a long-standing controversy. A HiPIP (high potential iron-sulfur protein) encoded by *iro *has been postulated to be the first electron acceptor from Fe(II) [44,45]. However, transcriptional studies of *iro *in *A. ferrooxidans *ATCC33020 suggested that it may be involved in sulfur oxidation. In our analysis of the type strain, *iro *(AFE2732) was found to be associated with the petII gene cluster thought to be involved in sulfur oxidation [46,47], thus making it unlikely that Iro is the key iron-oxidizing enzyme.

##### 2.2.2 Aerobic oxidation of reduced inorganic sulfur compounds (RISCs)

Genes encoding enzymes and electron transfer proteins predicted to be involved in the oxidation of reduced inorganic sulfur compounds (RISCs) were detected in the genome (Figure 3b). The oxidative and electron transfer pathways for RISCs are more complicated than those for Fe(II) oxidation, making their prediction and elucidation more difficult [48]. To add further complication, some steps occur spontaneously, without enzymatic catalysis. Previous experimental studies in various strains of *A. ferrooxidans *detected several enzymatic activities involved in the oxidation of RISCs [1,28], but some of these activities had not been linked to specific genes. Based on genome analysis, some of these missing assignments are predicted and also some novel genes involved in the oxidation of thiosulfate, sulfide, and tetrathionate are suggested.

Experimentally validated components of RISC metabolism include: the *pet-II *operon (AFE2727-31) and alternative quinol oxidases of the *bd *(AFE0954-5) and *bo*_3 _families (AFE0631-4) [7,8]; a sulfide/quinone oxidoreductase encoded by *sqr *(AFE0267) suggested to be involved in the oxidation of sulfide to sulfur [49,50]; and a tetrathionate hydrolase encoded by *tetH *(AFE0029) thought to be involved in the oxidation of tetrathionate [51].

The two homologs of *doxDA *(AFE0044; AFE0048) present in the genome are predicted to encode a thiosulfate/quinone oxidoreductase. Both appear to be a fusion of the separate *doxD *and *doxA *genes that are found in other organisms such as *A. ambivalens *[52,53]. Both are located in a major gene cluster composed of two divergent gene clusters. The first region (AFE0050-47) encodes a protein with TAT-signal peptide (IPR006311, TIGR01409), a periplasmic solute-binding protein, the first *doxDA *gene, and a conserved hypothetical protein. The second region (AFE0046-42) encodes a conserved hypothetical protein, a rhodanese enzyme that splits thiosulfate into sulfur and sulfite [54], the second copy of *doxDA*, a periplasmic solute-binding protein, a second copy of a gene encoding a protein with TAT-signal peptide (IPR006311, TIGR01409), and a gene encoding a putative carboxylate transporter. We have detected a similar organization in the *Gluconobacter oxydans *genome.

Five genes, predicted to encode thiosulfate sulfur transferase (rhodanese) proteins (AFE2558, AFE2364, AFE1502, AFE0529 and AFE0151) are dispersed in the genome [55] but their roles in sulfur oxidation remain to be firmly established. Notably, some of these predictions are based on the presence of the rhodanese PFAM00581 motif associated with phosphatases and ubiquitin C-terminal hydrolases, in addition to sulfur oxidation. Genes were not detected for several enzymatic functions that have been experimentally demonstrated in other strains of *A. ferrooxidans *including the sulfur dioxygenase that oxidizes persulfide-sulfur to sulfite in *A. ferrooxidans *strain R1 [1,56] and the sulfite oxidase that oxidizes sulfite to sulfate in *Ferrobacillus ferrooxidans *[1,57].

##### 2.2.3 Hydrogen and formate utilization

**Figure 4:**
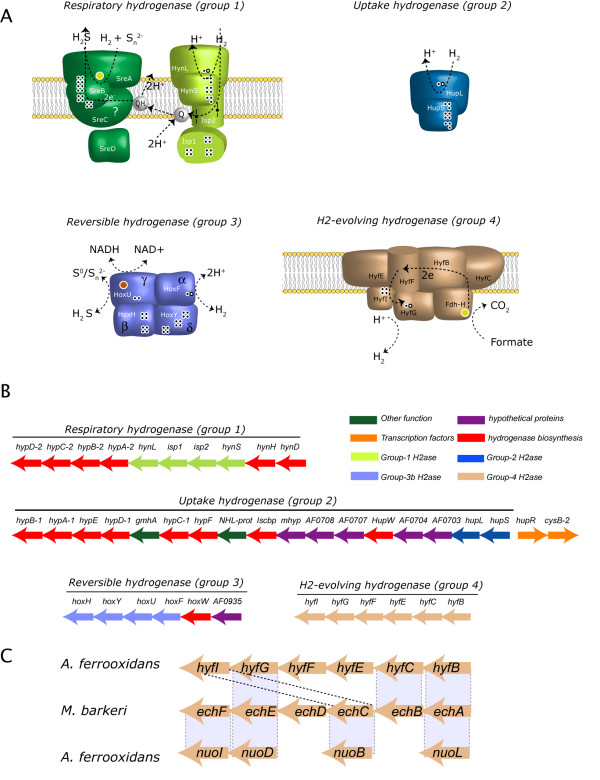
**Diversity and genomic organization of predicted hydrogenases**. A) Schematic representation of the four predicted types of hydrogenase. B) Organization of the predicted operons encoding the four types of hydrogenase. C) Schematic representation of similarity between the group 4 hydrogenase genes in *M. barkeri *with the *A. ferrooxidans *group 4 hydrogenase (above) and NADH dehydrogenase subunits (below).

Hydrogen utilization has been demonstrated experimentally in *A. ferrooxidans *ATCC 23270 [9] and a group 2 hydrogenase from *A. ferrooxidans *ATCC 19859 has been characterized [58], but there were no previous reports describing the hydrogenase genes and their genetic organization or their potential diversity. The *A. ferrooxidans *genome encodes four different types of hydrogenases based on the 2001 classification by Vignais et al. [59] (Figure 4, Additional file 3). Group 1 [NiFe]-hydrogenases are membrane-bound respiratory enzymes that enable the cell to use molecular hydrogen as an energy source. *A. ferrooxidans *has both the predicted structural (AFE3283-86) and the maturation-related genes (AFE3281-2; AFE3287-90) required for production of a functional respiratory hydrogenase of this type. In addition, the small subunit of this predicted complex has the characteristic TAT-signal peptide used to target the full heterodimer to the periplasmic space [60]. The genomic arrangement of the structural genes (*hynS-isp1-isp2-hynL*) is identical to that found in a thermoacidophilic archaeon (*Acidianus ambivalens*), a hyperthermophilic bacterium (*Aquifex aeolicus*), a denitrifying bacterium (*Thiobacillus denitrificans*), and two phototrophic sulfur bacteria (*Thiocapsa roseopersicina *and *Allochromatium vinosum*). Like *A. ferrooxidans*, all of these bacteria are chemoautotrophs that live in extreme environments, use inorganic energy sources, and have an active sulfur metabolism that oxidizes and reduces inorganic sulfur compounds [61].

*A. ferrooxidans *also encodes a group 2 cytoplasmic uptake [NiFe]-hydrogenase (AFE0701-2). Group 2 hydrogenases are induced during nitrogen fixation to utilize the molecular hydrogen generated [62]. The cyanobacterial-like hydrogenase in *A. ferrooxidans *exhibits the characteristic features of uptake hydrogenases as determined by EPR and FTIR [63]. Divergently oriented from the group 2 hydrogenase gene cluster is a predicted σ_54_-dependent hydrogenase transcriptional regulator (*hupR*) (AFE0700). HupR together with a histidine kinase forms part of a two-component regulatory system in *R. eutropha *[64], but the histidine kinase appears to be absent from the *A. ferrooxidans *genome. Despite that, HupR is able to activate transcription in the non-phosphorylated form [65-67], indicating that HupR is still able to regulate transcription of the group 2 hydrogenase system in *A. ferrooxidans*.

Adjacent to the group 2 hydrogenase gene cluster and transcribed in the same direction is a predicted cysteine regulon transcriptional activator *cysB *(AFE0699). This is followed by a cluster of genes potentially involved in fermentation, including a predicted σ_54_-dependent transcriptional regulator and a group of *isc*-like genes (AFE0672-78). The latter gene group is thought to be involved in assembling the iron-sulfur cluster of the nitrogenase used in nitrogen fixation, thus suggesting a connection between hydrogen production by the group 2 hydrogenase and nitrogen fixation [68]. The close proximity of the fermentation gene cluster suggests an additional metabolic coupling with fermentative metabolism, perhaps as part of a σ_54 _regulatory cascade operating in anaerobic or microaerophilic conditions.

The third predicted hydrogenase encodes a sulfhydrogenase, a group 3b cytoplasmic, bidirectional, heterotetrameric hydrogenase. This hydrogenase, in association with other proteins, binds soluble cofactors such as NAD, cofactor 420, and NADP [59]. Domain analysis predicts an F420 binding site in the α subunit (large hydrogenase subunit; AFE0937) and NAD- and FAD-binding sites in the γ subunit (AFE0939). The predicted NAD-binding site suggests that *A. ferrooxidans *can use NADPH as an electron donor, as has been shown for *Pyrococcus furiosus *[69]. A possible role for this hydrogenase could be the recycling of redox cofactors using protons or water as redox counterparts, as has been suggested for *Alcaligenes eutrophus*, thus serving as an electron sink under high reducing conditions [66].

The gene organization and amino acid sequence of a six-gene cluster (AFE2149-54) (Figure 4c) shows significant similarity to the group 4 H_2_-evolving hydrogenase complex found in several organisms (e.g., *Methanococcus barkeri *[70]). In *M. barkeri*, this cluster encodes a six-subunit complex that catalyzes the energetically unfavorable reduction of ferrodoxin by H_2_, possibly driven by reverse electron transport. The reduced ferrodoxin produced then serves as a low-potential electron donor for the synthesis of pyruvate in an anabolic pathway [71]. Reverse electron flow for the production of NADH via the oxidation of Fe(II) in *A. ferrooxidans *has been shown to be driven by the proton motif force (PMF) across its membrane that results from the acidity of its environment [72]. The predicted activity of the group 4 hydrogenase complex may exemplify another where *A. ferrooxidans *exploits the natural PMF to generate reducing power and couple it to redox reactions.

Another possible role for the group 4 hydrogenase complex involves the oxidation of formate. Two clusters of three genes (AFE1652-4 and AFE0690-2) potentially encode a formate dehydrogenase complex consisting of a formate dehydrogenase accessory protein FdhD-1, a hypothetical protein, and a molybdopterin formate dehydrogenase. The second cluster is divergently oriented from a gene encoding a predicted σ_54_-dependent transcriptional regulator. It has been reported that this complex associates with a hydrogenase group 4 complex in *E. coli *to create a formate hydrogenase supercomplex [73]. We propose a similar model for *A. ferrooxidans*, thus offering a biochemical basis for its ability to oxidize formate [10].

##### 2.2.4 Anaerobic metabolism

Several strains of *A. ferrooxidans *have been reported to use electron acceptors other than O_2_, including the use of ferric iron for the oxidation of sulfur and hydrogen and the use of sulfur for the oxidation of hydrogen by *A. ferrooxidans *JCM 7811 [74]. In that strain, the reduction of ferric iron was accompanied by the increased expression of a 28 kDa c-type cytochrome that was suggested to be responsible for this activity [74]. The reduction of ferric iron during sulfur oxidation was also shown for the type strain ATCC 23270 [75]. However, a gene potentially encoding this cytochrome could not be identified in *A. ferrooxidans *ATCC 23270 [76]. A candidate iron reduction complex has been investigated in *A. ferrooxidans *AP19-3 by electrophoretic purification and enzymatic assays [77,78]. However, potential genes encoding this complex could not be detected in our genome analysis.

The use of sulfur as an electron acceptor was investigated in *A. ferrooxidans *NASF-1 where aerobically grown cells were found to produce hydrogen sulfide from elemental sulfur using NADH as electron donor via a proposed sulfur reductase [79]. However, the observed molecular weights of the subunits of this sulfur reductase do not correspond to those predicted from an analysis of the group 3b hydrogenase genes in the type strain genome, with the caveat that post-translational modifications could explain the differences in molecular weights. However, a gene cluster (AFE2177-81) was detected in the type strain that is predicted to encode a sulfur reductase enzyme with significant similarity of amino acid sequence and gene order to the cluster suggested to be responsible for sulfur reduction in *Acidianus ambivalens *[80]. We hypothesize that this enzyme could associate with the predicted group 1 hydrogenase to form a supercomplex, facilitating the use of hydrogen as an electron and energy source with sulfur serving as the final electron acceptor.

#### 2.3 Nitrogen metabolism

*A. ferrooxidans *can meet its nitrogen needs by either nitrogen fixation or ammonia assimilation. Diazotrophic growth of *A. ferrooxidans *was first demonstrated in early studies of acetylene reduction and ^15^N_2 _assimilation [15] and the structural genes for the nitrogenase complex were later sequenced [81-83].

##### 2.3.1 Ammonia uptake and utilization

The *A. ferrooxidans *genome contains genes predicted to be involved in ammonia uptake (*amt1*, *amt2 *and *amtB*; AFE2916, AFE2911, and AFE1922). *Amt1 *and *amt2 *are located in a gene cluster that includes a gene potentially encoding a class-I glutamine amidotransferase (AFE2917) that has been shown in other organisms to transfer ammonia derived from the hydrolysis of glutamine to other substrates. *GlnK-1 *(AFE2915) is also present in this cluster and is predicted to encode a P-II regulatory protein involved in the regulation of nitrogen metabolism in response to carbon and glutamine availability [84]. A *glnA *homolog (AFE0466) is predicted to encode a type I glutamine synthase that would permit the incorporation of ammonia directly into glutamine, completing the inventory of genes necessary for ammonia uptake and utilization.

##### 2.3.2 Nitrogen Fixation

A putative nitrogenase gene cluster (*nifH-D-K-fer1-fer2-E-N-X*; AFE1522-AFE1515) (Additional file 4) was previously reported in the type strain [68]. These genes potentially encode the nitrogenase complex and proteins involved in the synthesis of the nitrogenase MoCo cofactor. In other organisms, nitrogenase has been shown to be oxygen sensitive and its expression and activity are regulated at both the transcriptional and post-translational levels [84]. Divergently oriented from the *nif *operon is a cluster of genes involved in the regulation of nitrogenase activity. The first gene of this cluster is a putative σ_54 _response regulator (AFE1523). This is followed by the *draT *and *draG *genes (AFE1524, AFE1525) that encode a dinitrogen-reductase ADP-D-ribosyltransferase and a ADP-ribosyl-[dinitrogen reductase] hydrolase, respectively. These two are involved in the post-translational modulation of nitrogenase activity in response to ammonium and oxygen concentrations [84]. *NifA *(AFE1527) is also present in the same gene cluster. *NifA *potentially encodes an enhancer binding protein that, together with σ_54_, is involved in the transcriptional activation of the *nif *operon in response to the redox, carbon, and nitrogen status. This ensures that nitrogen fixation occurs only under physiological conditions that are appropriate for nitrogenase activity [85].

**Figure 5:**
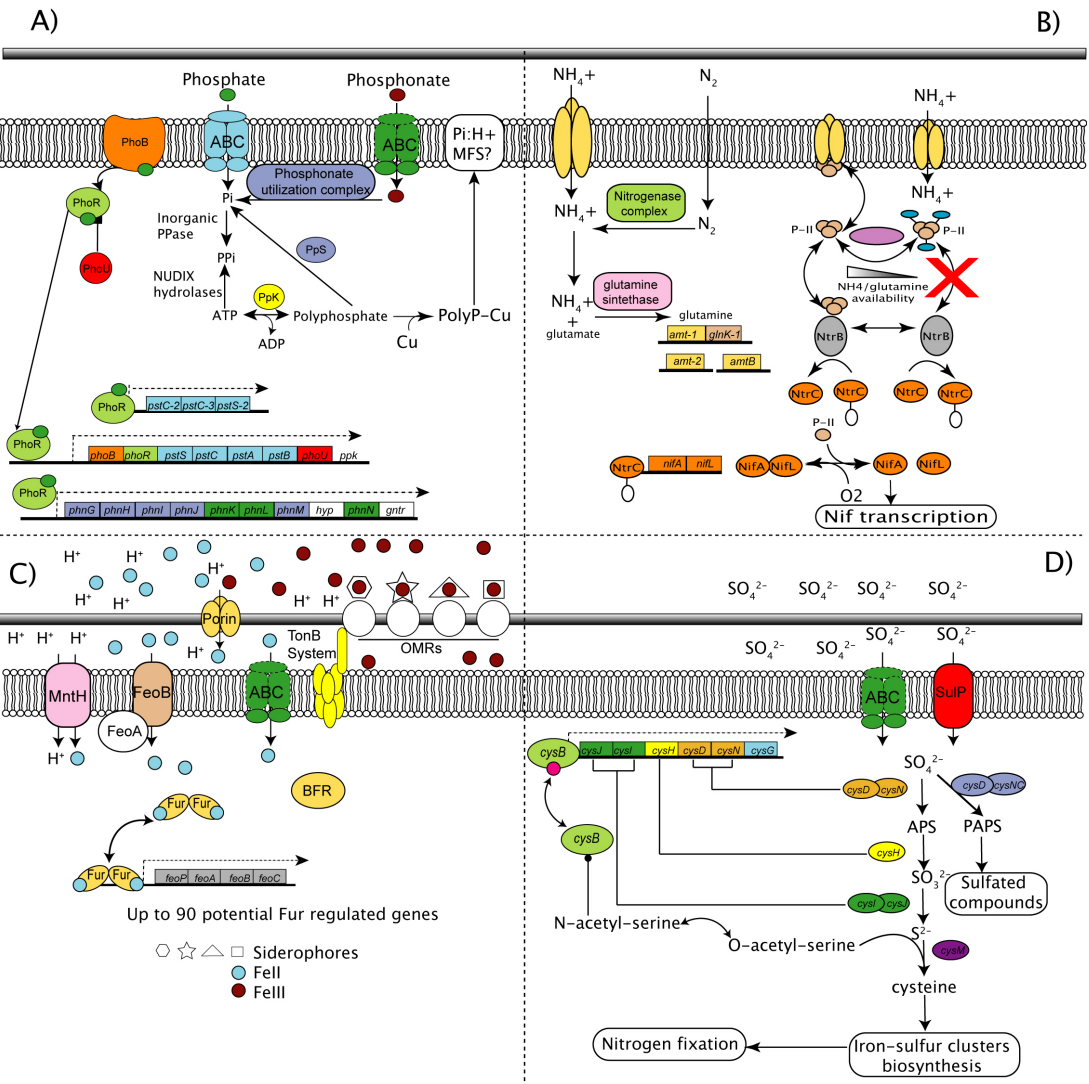
**Predicted regulatory models for inorganic ion uptake and assimilation**. A) Phosphate and phosphonate. B) Nitrogen and ammonia. C) Ferric and ferrous iron. D) Sulfate. Proteins and protein complexes are described in the text.

Using this genomic information, a gene network for the regulation of nitrogen fixation and ammonia uptake can be suggested for *A. ferrooxidans *that is consistent with similar models derived from other organisms (Figure 5) [84]. In this model, NifA (AFE1527) is the transcriptional activator of the nitrogenase operon and its expression is regulated by a two-component regulatory system encoded by *ntrB *and *ntrC *(AFE2902, AFE2901) that measure oxygen and nitrogen levels. These signals are integrated by the P-II proteins (*glnK-1*, AFE2915; *glnB-1*, AFE2462; *glnK-2*, AFE2240; and *glnB-2*, AFE0429) with additional metabolic signals, such as fixed carbon and energetic status [86]. Two additional copies of *ntrC *and *ntrB*, termed *ntrY *and *ntrX *(AFE0024, AFE0023) have been detected in the genome that could allow cross talk between the sensor/regulator pairs NtrY/X and NtrB/C, as described in *Azospirillum brasilense *[87]. The redundancy of the regulatory genes responsible for nitrogen fixation and assimilation suggests the presence of a flexible mechanism that is responsive to environmental changes.

### 3. Nutrient uptake and assimilation systems

*A. ferrooxidans *has 72 genes (2.23%) predicted to be involved in nutrient uptake (Additional file 3) whereas most heterotrophic γ-proteobacteria typically dedicate about 14% of their genome information to transport functions [88]. The potential substrates incorporated include phosphate, sulfate, iron, ammonia, organic acids, amino acids, and sugars. This repertoire, especially the low representation of predicted carbohydrate uptake systems, is a signature of obligate autotrophic bacteria [88].

#### 3.1 Inorganic ion assimilation

##### 3.1.1 Sulfate

A gene for a predicted sulfate permease (AFE0286) of the SulP family is present in the genome adjacent to a potential carbonic anhydrase gene (AFE0287). This linkage has been observed in many bacteria [89], suggesting that the gene pair forms a sulfate/carbonate antiporter system. Sulfate taken up from the environment is thought to be reduced to sulfide for cysteine biosynthesis by a group of genes belonging to the *cys *regulon [68].

##### 3.1.2 Phosphate

Previous investigations of phosphate metabolism in *A. ferrooxidans *provided evidence for a phosphate starvation response [90] and for a relationship between polyphosphate degradation and heavy metal resistance and efflux [91]. However, we still lacked a comprehensive understanding of all the potential components involved in phosphate metabolism, as well as their integration and regulation. Our genome analysis identified a complete repertoire of the genes necessary for phosphate uptake by the high affinity Pst-transport system. These predicted genes are arranged in two similar clusters. The first cluster (AFE1939-41) includes two genes (*pstC-1 *and *pstC-2*) that encode a phosphate permease and a third gene (*pstS-1*) that encodes a periplasmic phosphate binding protein. The second gene cluster (AFE1441-1434) includes a gene encoding a exopolyphosphatase (*ppx*, AFE1441) previously described to be involved in heavy metal resistance and efflux [91], *phoU *(AFE1440) predicted to encode a phosphate transport regulatory protein, *pstB *encoding an ATP binding protein, and *pstA *coding for the permease component. In addition, there are genes encoding a third homolog of the phosphate permease PstC-3, a second homolog of the periplasmic phosphate binding protein PstS-2, and the two-component response regulator PhoR/PhoB.

The predicted phosphonate utilization gene cluster (AFE2278-86) contains genes for C-P cleavage and an ATP-binding protein for the ABC phosphonate transport system. In spite of the experimental evidence reported about the utilization of phosphonate in this bacterium [92], the typical permease subunit that is required to complete phosphonate uptake was not found in the genome.

The genome does contain a gene (AFE1876) for a predicted polyphosphate kinase (Ppk) involved in polyphosphate storage. It has been suggested to be part of a *pho *regulon whose expression is activated during phosphate starvation [89] and in response to heavy metal toxicity [91].

##### 3.1.3 Iron

Genomic evidence indicates that *A. ferrooxidans *relies on diverse standard iron uptake mechanisms to obtain both Fe(II) and Fe(III) (93). The type strain has candidate genes (AFE2523-AFE2525) potentially encoding the FeoABC Fe(II) inner-membrane transport system and an NRAMP dual Mn(II)/Fe(II) MntH-like transporter (AFE0105). Previously reported gene context analysis indicated that *feoA, feoB*, and *feoC *form part of an iron-regulated operon, along with an ORF (AFE2522) encoding a putative porin (designated *feoP*) that could facilitate entrance of Fe(II) into the periplasm [93].

*A. ferrooxidans *is typically confronted with an exceptionally high concentration of soluble iron in its acidic environment, as high as 10^-1^M compared to 10^-16 ^M in typical neutrophilic environments. This raises questions as to the mechanisms it uses for iron assimilation and homeostatic control of internal iron concentrations. Given the abundance of both Fe(II) and Fe(III) in its environment, *A. ferrooxidans *has a surprisingly large number of iron uptake systems, including eleven distinct putative genes encoding TonB-dependent outer membrane receptors (*tdr*) for high affinity uptake of siderophore-chelated Fe(III) (*tdrA*, AFE2935; *tdrC*, AFE1483; *tdrD*, AFE1492; *tdrE*, AFE2040; *tdrF*, AFE2998; *tdrG*, AFE2302; *tdrH*, AFE2298; *tdrI*, AFE2292; *tdrJ*, AFE2288; *tdrK*, AFE0763; *tdrL*, AFE3229). Also, it has a number of copies of all the accessory genes needed to transport iron into the cytoplasm, including seven different copies of the energy transduction genes *tonB *(AFE3002, AFE2304, AFE2301, AFE2275, AFE2268, AFE1487, AFE0770) and *exbB *(AFE3003, AFE2299, AFE2273, AFE2270, AFE1485, AFE0768, AFE0485) and six copies of *exbD *(AFE3004, AFE2300, AFE2269, AFE1486, AFE0769, AFE0486), as well as the genes encoding two different ABC iron transporters (AFE1489-AFE1491, AFE1493-AFE1495). No genes were detected that might be involved in standard mechanisms of siderophore production. However, its multiple siderophore uptake systems suggest that it is nonetheless capable of living in environments where iron is scarce (perhaps at higher pH values) and in which other organisms capable of producing siderophores are present.

For Fe(III) uptake, all the genes involved are organized in seven gene clusters, some of which include additional gene functions [22]. One cluster encodes a complete suite of proteins necessary for Fe(III) uptake (AFE1482-AFE1495) that includes not only two outer membrane receptors (OMRs) of different predicted siderophore specificities, but also three different ABC solute-binding proteins with affinity for iron and molybdenum and may be a dedicated iron-molybdenum transport system that is present in a genomic island [94]. This predicted operon also includes a putative gene (*gloA*, AFE1482) predicted to encode a globin-like protein that has been suggested to be an oxygen sensor regulating the expression of Fe-Mo uptake [94]. GloA is also associated with an upstream Fur box, indicating possible regulation via the master iron regulator Fur [21].

#### 3.2 Carbon compound uptake

##### 3.2.1 Amino acids

Among the predicted nutrient transport genes in the *A. ferrooxidans *genome are five amino acid permeases of unknown specificity (AFE2659, AFE2457, AFE1782, AFE0719, and AFE0439) (the same number as found in the chemolithoautotroph *T. crunogena*) and one complete ABC system for dipeptide uptake (AFE2987-92). The addition of leucine to solid media has been reported to improve the yield of *A. ferrooxidans *ATCC 33020 during the first ten days of growth, whereas the addition of cysteine or methionine inhibits growth [95]. More recently, the addition of minimal concentrations of glutamate to liquid media was found to accelerate the growth rate of *A. ferrooxidans *ATCC 23270 (Omar Orellana, personal communication).

##### 3.2.2 Carbohydrate uptake

The suite of genes for carbohydrate transport appears to be limited, as has been found in most obligate autotrophs (e.g., *T. denitrificans *[96], *T. crunogena *[88], *M. capsulatus *[97], *N. europea *[98], and *N. oceanii *[99]). This suite of predicted genes includes two outer membrane carbohydrate selective porins of the OPRB family (AFE2522, AFE2250), one carbohydrate transporter of unknown specificity (AFE2312) that is related to the major facilitator superfamily (PF00083, PS50850) and very similar to xylose and galactose proton symporters [100], and an MFS transporter (AFE1971) with marginal similarity to sugar/nucleoside symporters. It also includes genes for an incomplete PTS system for carbohydrate uptake (AFE3018-23) potentially encoding EII-A, a kinase/phosphatase HprK, an ATPase, an IIA component, a phosphocarrier protein Hpr, and a phosphoenolpyruvate phosphotransferase. However, we could not identify a gene encoding the IIC sugar permease component, thus making it unlikely that *A. ferrooxidans *has a functional sugar-transporting PTS system. Instead, we suggest that this PTS system could be involved in molecular signaling as part of a regulatory cascade involving RpoN, as described in other proteobacteria [101]. In this model, a decrease of fixed carbon leads to low levels of phosphoenolpyruvate and cyclic-AMP that in turn maintain most PTS proteins in the dephosphorylated form. This promotes the utilization of glycogen as a carbon source to replenish the phosphoenolpyruvate levels, thus restoring the levels of phosphorylated PTS proteins [102].

### 4. Central carbon metabolism

**Figure 6:**
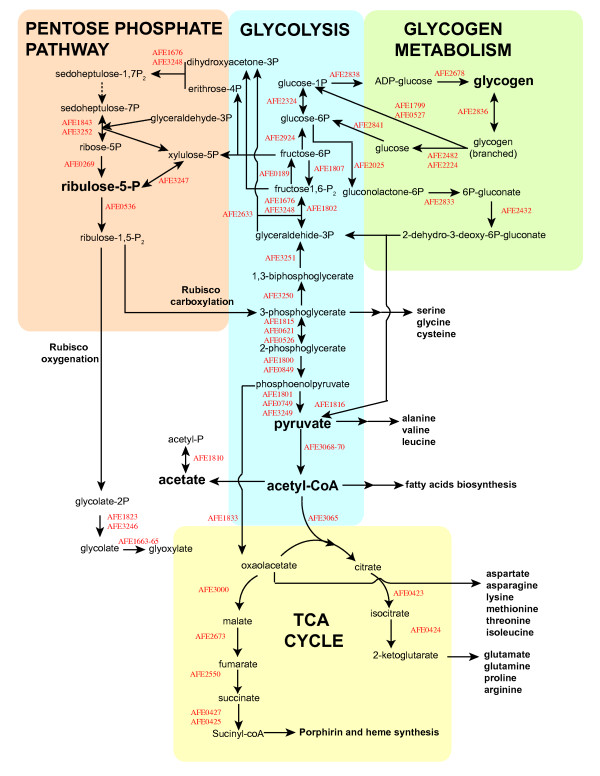
Predicted pathways (pentose phosphate pathway, glycolysis, glycogen and interrupted TCA cycle) for central carbon metabolism.

It has been shown in many organisms that the 3-phosphoglyceraldehyde generated by CO_2 _fixation via the Calvin cycle enters the Embden-Meyerhof-Parnass pathway, thus providing fixed carbon that can be channeled in either of two directions: for glycogen biosynthesis and storage, or to provide carbon backbones for anabolic reactions. The genes predicted for these two pathways in *A. ferrooxidans*, together with their reactions and potential interconnections with other biosynthetic pathways, are shown in Figure 6.

#### 4.1 Carbon storage and utilization

The genome also contains genes predicted to encode the five enzymes required for glycogen biosynthesis from fructose-6P. As has been shown in other organisms, glucose-1P-adenylyltransferase (*glgC*, AFE2838) is predicted to synthesize ADP-glucose. A specific glycogen synthase (*glgA*, AFE2678) would then transfer the glucosyl moiety of ADP-glucose to a glycogen primer to form a new 1,4-glucosidic bond. Subsequently, a branching enzyme (*glgB*, AFE2836) is predicted to catalyze the formation of branched 1,6-glucosidic linkages.

The carbon stored in glycogen is thought to be released by glucan phosphorylase (*glgP1*, AFE1799; *glgP2*, AFE0527), thus regenerating glucose-1P from the non-reducing terminus of the 1,4 chain. The pathway for the conversion of glucose-6P to 2-dehydro-3-deoxy-gluconate is also predicted to be present, except for the last step that replenishes the levels of pyruvate and 3P-glycerate. In addition, a gene encoding phosphoribulokinase was not detected, thus suggesting that either alternate genes encode the missing functions or else that *A. ferrooxidans *regenerates pyruvate and 3P-glycerate from stored glycogen by alternate pathways. Expression data obtained from *A. ferrooxidans *growing with sulfur and iron as energy sources have shown that genes involved in glycogen synthesis and utilization are differentially expressed [103]. Specifically, growth in sulfur-containing media preferentially activates genes involved in glycogen biosynthesis, whereas growth on iron-supplemented media upregulates genes involved in glycogen breakdown. This suggests that *A. ferrooxidans *channels fixed carbon to glycogen when sulfur is available as an energy source and uses glycogen as a reserve carbon donor when iron is the energy source.

#### 4.2 Carbon backbone formation

The genome contains three genes (AFE1802, AFE1676 and AFE3248) that are predicted to encode fructose biphosphate aldolase (EC. 4.1.2.13), the enzyme that catalyzes the formation of fructose-1,6-bP. The interconversion of fructose-1,6-bP to fructose-6P in most heterotrophic bacteria is carried out by fructose biphosphatase and phosphofructokinase enzymes. In *A. ferrooxidans*, a gene encoding a fructose biphosphatase enzyme was found (AFE0189) that we suggest allows a direct flux of fixed carbon to glycogen storage. A potential phosphofructokinase candidate gene (AFE1807) was also found, a member of the PfkB family of sugar kinases (cd01164). It is located near putative genes involved in glycolysis/glyconeogenesis (e.g., phosphoglycerate mutase and phosphoenolpyruvate synthase), thus generating a bidirectional metabolic path for the utilization/generation of glycogen.

Putative genes for all the enzymes involved in the conversion of glyceraldehyde-3-P to pyruvate and acetyl-coA, as well as for the citric acid (TCA) cycle, were detected with the exception of genes encoding the E1-3 subunits of α-ketoglutarate dehydrogenase. Thus, the TCA cycle is incomplete, as has been described in a number of obligate autotrophic bacteria and archaea – a likely hallmark of this lifestyle [104].

### 5. Heavy metal resistance

Bioleaching microorganisms, such as *A. ferrooxidans*, typically live in environments that have high concentrations of soluble heavy metals (e.g., arsenic, mercury, and silver), as well as unusually high concentrations of potentially toxic metals (e.g., copper and iron). This has prompted numerous studies of the mechanisms employed by *A. ferrooxidans *for metal resistance [105]. In contrast to the genomic perspective presented herein, those investigations were conducted on multiple strains and thus do not provide a coherent view of the repertoire of heavy metal resistance genes present within one strain.

Our genome analysis confirmed the presence of a divergent gene cluster (AFE2857-60) previously identified as involved in arsenic resistance. The cluster includes genes encoding an arsenate reductase (ArsC), the arsenate repressor (ArsR), the divergently-oriented arsenate efflux pump (ArsB), and a hypothetical protein (ArsH). The *arsCRB *gene cluster was shown to confer resistance to arsenate, arsenite, and antimonium in *E. coli*, but the function of *arsH *is unknown [106,107].

Mercury resistance has been investigated in several strains of *A. ferrooxidans *[108-110]. Genome analysis of the type strain identified three genes potentially encoding the well-described Mer components, i.e., the repressor accessory protein (MerD, AFE2483), the mercury reductase (MerA, AFE2481), and the mercuric ion transporter (MerC, AFE2480). Four candidate genes potentially encoding members of the family of MerR-like transcriptional regulators were also found (AFE2607, AFE2509, AFE1431, and AFE0373).

The *A. ferrooxidans *genome also contains several genes (Additional file 3) predicted to be part of heavy metal tolerance systems [111], including genes for the copCD copper extrusion system, ten clusters of genes predicted to belong to the resistance-nodulation-cell division (RND) family of transporters, three genes encoding cation diffusion facilitator (CDF) proteins, three genes encoding copper translocating P-type ATPases, and two genes encoding other P-type ATPases of unknown specificity. These genome-based predictions offer new opportunities for experimental validation of heavy metal resistance in *A. ferrooxidans *and also provide new markers for detecting similar genes in other microorganisms

### 6. Extrusion of toxic organic compounds

The ability to extrude toxic organic compounds is widespread, and our inspection of the *A. ferrooxidans *genome suggests that this bacterium is well equipped to deal with toxic organic molecules. Its genome contains a gene predicted to encode the toluene tolerance protein TtgD (AFE1830) as well as a cluster of proteins often associated with toluene resistance that includes a Tol-Pal-associated acyl-CoA thioesterase (AFE0063) and TolBARQ (AFE0064-67).

The genome also includes a predicted complete ABC gene cluster (AFE0158-63) involved in drug extrusion that has significant similarity to the toluene ABC resistance proteins reported in other organisms. Resistance to toluene/xylene and related aromatic hydrocarbons and organic solvents may be needed by *A. ferrooxidans *when growing in runoff from coal wastes where it might encounter aromatic hydrocarbons [112] or in bioleaching operation heaps that are irrigated with recycled water containing carboxylic acids and other organic compounds from solvent extraction operations [113].

An alternative hypothesis for the role of drug-related compound extrusion mechanisms present in microbes associated with biogeochemical cycles has been proposed [114]. A homolog of TolC has been shown in *Shewanella oneidensis *MR-1 to excrete anthraquinone-2,6-disulfonate (AQDS) that is used as an extracellular electron shuttle. It has been proposed that AQDS may be particularly important to transfer electrons from cells embedded in the interior of biofilms to reduce Fe(III) present in the solid substrate to which the biofilm is attached. It is possible that a similar mechanism may be used by *A. ferrooxidans *in the reverse process, namely, to convey electrons from the oxidation of Fe(II) present in solid minerals to cells not in contact with the substrate.

Two additional ABC systems potentially involved in drug extrusion are also predicted in the genome, each associated with a HlyD secretion protein family (AFE2861-64, AFE1603-7). The first is directly downstream from the *ars *genes; the second cluster may have originated through lateral gene transfer since it is flanked by truncated transposases and hypothetical genes and it also exhibits anomalous G+C content.

*A. ferrooxidans *may also be resistant to some antibiotics due to the presence of a two-gene cluster (AFE1977-78) potentially encoding a fosfomidocyn resistance protein and a TonB-family protein, respectively, and also a gene potentially encoding an AmpG permease protein (AFE1961).

### 7. Stress responses

For aerobically growing bacteria, the autooxidation of oxidases in the respiratory chain is the main source of endogenous reactive oxygen species (ROS). Increased levels of ROS can also result from exposure to redox active metals, including iron. Aerobic biomining microorganisms such as *A. ferrooxidans *that thrive in iron-rich environments are thus expected to be well equipped to deal with disturbances in oxidant-antiooxidant balance. Surprisingly, an unexpectedly low number of genes encoding known ROS detoxification functions were identified in the genome. These genes include a Mn-superoxide dismutase encoded by *sodA *(AFE1898), two non-identical copies of *ahpC*-like (AFE1468, AFE0985) and *ahpD*-like (AFE02014, AFE1814) members of the alkylhydroperoxidase family, and *nox*, (AFE1803) potentially encoding a NADH oxidase (FAD-dependent pyridine nucleotide-disulfide oxidoreductase family protein). This latter is considered to be important in oxygen scavenging in anaerobes because of its potential to reduce oxygen to water [115]. No genes coding for known catalases were detected.

On the other hand, *A. ferrooxidans *is predicted to have a complete set of components needed for non-enzymatic neutralization of ROS. This mechanism maintains high levels of low molecular weight thiols in the cytoplasm that, in combination with specific disulfide reductases, provide a reducing intracellular environment and maintain the thiol/disulfide balance of other molecules (unpublished results). Seven distinct thioredoxins (*txr*; AFE2867, AFE2848, AFE2590, AFE2362, AFE1979, AFE0657, AFE0047) and one thioredoxin disulfide reductase (*trxB*, AFE0375) are present. Also present are the genes of the glutathione system necessary for glutathione-tripeptide synthesis from the amino acids L-cysteine, L-glutamate, and glycine (*gshA*, AFE03064; *gshB*, AFE03063), four distinct glutaredoxins (*gxr*; AFE3038, AFE2449, AFE2263, AFE0367), and the glutathione reductase *gorA *(AFE0366).

In some bacteria, when basic protection is not sufficient, e.g., when sudden large increases in ROS occur, rapid global responses are induced to cope with the oxidative stress [116]. Often survival during the period of stress is aided by the simultaneous employment of multiple strategies. The strategies predicted to be available to *A. ferrooxidans *include repair of oxidative damage (e.g., *nfo*), bypassing of damaged functions (e.g., resistant isozymes *acnA, fumC*), and the exclusion of oxidative stress agents (e.g., *acrAB *multidrug efflux pump). Typically, many of these functions are coordinately regulated in response to superoxide by the SoxRS two-component regulator, and in response to peroxide by OxyR in Gram-negative bacteria or by PerR in Gram-positive bacteria. *A. ferrooxidans *lacks *oxyR*, *soxR*, and *soxS *orthologs, but has a Fur family regulator similar to PerR (AFE1467). The role of PerR in the control of *A. ferrooxidans *inducible stress response has not been investigated, but could include regulation of the divergently-transcribed AhpC family peroxidase (AFE1468). This arrangement is conserved in other microorganisms [117].

Other antioxidant defenses that are not controlled by the major oxidative stress regulators include the DNA repair enzyme endonuclease III (*nth*, AFE2682), glycoylases (*mutM*, AFE2758; *mutY*, AFE3015), DNA polymerase I (*polA*, AFE3094), recombinase protein A (*recA*, AFE0932), and other defenses including a peptide methionine sulfoxide reductase (*msrAB*, AFE2946-45) and a molecular chaperone (*hlsO*, AFE1408).

### 8. Flagella formation and chemotaxis

Conserved *fla *or *fla*-related genes that could encode flagella were not identified in the genome, nor were *che *genes that encode classic chemotaxis functions. These observations conflict with Ohmura et al. (1996) [118] who proposed that the formation of flagella was a major factor mediating the adhesion of *A. ferrooxidans *ATCC 23270 to solid sulfur surfaces. This discrepancy could be explained if the *fla *genes have been lost in the particular culture used for sequencing. Since flagella genes are encoded in a multigene operon in many bacteria, their complete loss might require only one or a small number of excision events. In contrast, the multiple *che *genes are usually widely dispersed in bacterial genomes and their collective loss in *A. ferrooxidans *ATCC 23270 would presumably require multiple excision events. Alternative hypotheses to explain this discrepancy include (i) contamination of the *A. ferrooxidans *ATCC 23270 culture used by Ohmura et al. (1996) by a flagella-bearing microorganism, and (ii) significant differences between the culture used by Ohmura et al. (1996) and that used for our genome sequencing despite their identical designation (ATCC 23270).

### 9. Adhesion and biofilm formation

For mineral-associated bacteria, adhesion and biofilm formation are critical steps for colonization and subsequent mineral solubilization [119]. Cell surface structures such as pili have been shown to play a critical role in auto-aggregation of microbial cells involved in biogeochemical processes [120]. *A. ferrooxidans *contains several gene clusters potentially involved in the formation of a type IV pilus (AFE0967-73, AFE0735-39, AFE0416, AFE0183-6, and AFE0006-7). Some of the relevant genes identified include those for the σ_54_-dependent transcriptional regulator *pilR *(AFE0185) and for the signal transduction histidine kinase *pilS *(AFE0184). In addition, candidate *tad *(tight adherence) genes (AFE2699-AFE2708) were also detected (Additional file 5). These genes are responsible for the secretion and assembly of bundled pili. In *A. actinomycetemcomitans*, they are essential for tight adherence, autoaggregation, and pili formation during colonization of dental surfaces [121]. They are also present in *Thiomicrospora crunogena *[88], a RISCs-oxidizing, chemoautotrophic bacterium found in thermal vents. The multiple copies of genes for pili biosynthesis and adhesion in *A. ferrooxidans *could enable attachment and colonization on various mineral surfaces, such as pyrite, chalcopyrite, and solid sulfur. The redundancy of related regulatory genes could allow *A. ferrooxidans *to respond successfully to environmental changes.

Genes involved in quorum sensing that were previously identified and characterized include those that encode the classical autoinducer-binding transcriptional regulator LuxR (AFE1997) and the autoinducer synthesis protein LuxI (AFE1999) [122]. In addition, a second route for the production of homoserine lactones using the act system was predicted based on the presence of a gene encoding an acyltranferase (*act*, AFE2584) that was shown to be involved in the production of homoserine lactones of C14 length [123].

A five gene operon, containing *luxA*-*galE*-*galK*-*pgm*-*galM*, was assigned gene numbers AFE1341-45, respectively. This operon has been proposed to be involved in the formation of extra-cellular polysaccharide (EPS) precursors via the Leloir pathway [100]. *GalU *(AFE0445) and *galT*-like (AFE1237) have also been predicted to form part of the Leloir pathway and genes *rfbA*, *B*, *C *and *D *(AFE3295, AFE0441, AFE3294 and AFE0442, respectively) have been proposed to be involved in the biosynthesis of the EPS precursor dTDP-rhamnose. These groups of genes have been postulated to be involved in biofilm formation in *A. ferrooxidans *and their patterns of transcription were characterized in growth media with and without organic carbon supplementation [100].

### 10. Genetic transfer

A region of the genome (AFE1013-AFE1387, Figure 2) is enriched (84% versus 54% for the rest of the genome) in putative genes encoding hypothetical proteins, genes for DNA metabolism and sequences related to mobile elements such as transposases, plasmids, and bacteriophage (phage), and pseudogenes. The presence of site-specific recombinases and phage integrases in this region, as well as in other regions such as AFE2397-99, AFE0833-35 and AFE0507-9, indicates that *A. ferrooxidans *has been the target of phage infection. Although no phages are currently known to infect *A. ferrooxidans*, this finding suggests that further searching might be fruitful. Such phage could facilitate study of the mechanisms of viral infection in extremely acidic conditions, as well as serve as useful transducing agents for the genetic manipulation of *A. ferrooxidans*, as have been shown for the acidophilic archaeon, *Sulfolobus *spp. [124,125].

The genome contains clusters of genes whose sequence and gene order show significant similarity to both the Trb system of the Ri plasmid from *Rhizobium rhizogenes *and the Ti plasmid from *Agrobacterium tumefaciens *[126]. Most of these predicted genes potentially encode structural proteins of the type IV secretion system involved in conjugative DNA transfer. However, missing from the genome are the *trbC*, *trbH*, and *trbK *genes that encode an inner membrane lipoprotein, a pili structural protein, and a protein involved in plasmid immunity, respectively. Notably, one of two copies of a *trbG*-like gene is located within a highly conserved cluster in a position usually occupied by *trbH*, and it may assume the role of this missing gene. The absence of critical components of the conjugation system suggests that *A. ferrooxidans *ATCC 23270 has lost the capacity to carry out conjugation via the Trb mechanism. The question arises as to the origin of the Ti plasmid-like sequences in *A. ferrooxidans*. One possibility is that it was acquired from an *Agrobacterium*-related microorganism or an ancestor of such through conjugation. *A. ferrooxidans *and a free-living or plant root-associated *Agrobacterium *might share the same environment at the interface of acidic drainages and anaerobic soils/water.

Ten proteins predicted to be involved in plasmid stability and maintenance are present in the genome. This discovery, coupled with the detection of an extensive suite of predicted conjugation-related genes, provides additional evidence that *A. ferrooxidans *was capable of undergoing conjugation. Even though no natural conjugation partners are known, conjugation between *E. coli *and *A. ferrooxidans *has been achieved in the laboratory. The frequency of detectable marker transfer has been very low [95,127,128], and must be increased before this technique can be used for widespread genetic manipulation of *A. ferrooxidans*. Our finding of conjugation-related genes could stimulate further attempts.

Forty-one IS elements were identified, of which thirty-three could be classified as members of nine families according to the scheme of Mahillon and Chandler [129] (Additional file 3). The largest groups, designated here as ISafe3 (8 copies) and ISafe4 (3 copies), belong to the IS110 and IS3 families, respectively [129]. ISafe1, which is associated with phenotypic switching in *A. ferrooxidans *ATCC 19859 [130], was not detected in the genome of the type strain. Two non-identical copies of a Tn5468 transposon (family Tn7-like) were detected, each containing *tnsABCDorf5 *(AFE1201-AFE1205, AFE3199-95). The first copy is embedded in a suite of genes encoding hypothetical proteins; the second is associated with the *atp *operon and the *glmSU *as described for *A. ferrooxidans *ATCC 33020 [131].

### 11. Predicted osmotic balance and potential pH tolerance mechanisms

Acidophiles exhibit functional and structural properties that allow them to survive and proliferate in extremely acidic environments (pH 3 or below) [reviewed in 132]. These include: a) impermeable cell membranes (mostly in archaea); b) selective outer membrane porins; c) the generation of positive internal potential (Δψ) to create a chemosmotic barrier inhibiting proton influx; and d) the removal of excess internal protons by active proton pumping.

A putative gene (*omp40*, AFE2741) was identified that had significant similarity to an outer membrane porin found in *A. ferrooxidans *strain ATCC 19859 (133, 134). A large external, positively-charged loop has been predicted in Omp40 that may control pore size and ion selectivity at the porin entrance and may constitute a potential proton barrier [133,134].

In addition, the following related functions were predicted (Additional file 3): several potassium transporters including one K+ channel, one K+ uptake protein and one K+ efflux transporter; two copies of an ABC potassium import system that could be involved in the generation of a positive internal potential inhibiting proton influx; four Na/H+ antiporters and two proton P-type ATPases that could extrude excess internal protons. These predictions suggest specific areas for future experimental validation.

## Conclusion

**Figure 7:**
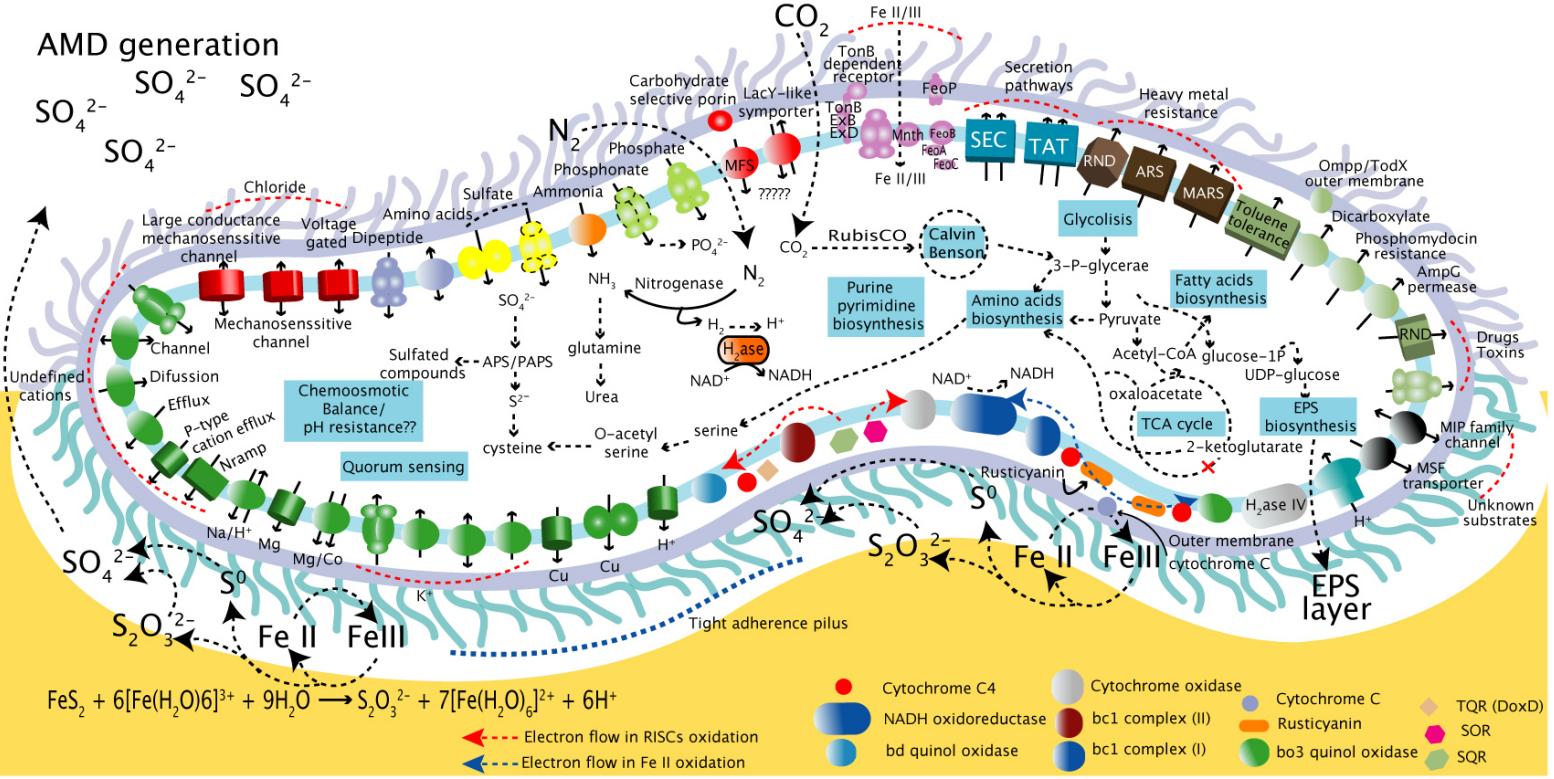
**Whole-cell model *for A. ferrooxidans *ATCC 23270**. Genome-based model of the cellular metabolism of *A. ferrooxidans *including predicted transport systems; chemolithoautotrophic components; carbon, nitrogen and sulfur metabolism; and biogeochemical cycling.

• Bioinformatics analysis of the complete genome of the type strain of *A. ferrooxidans *(ATCC 23207) provides a valuable platform for gene discovery and functional prediction that is especially important given the difficulties in carrying out standard genetic research in this microorganism. The models presented herein should facilitate the design and interpretation of future experiments and enable the experimental investigator to focus on important issues.

• An analysis of the genome of the type strain provides a coherent view of the gene content and metabolic potential of this species (Figure 7).

• Metabolic models support the key capabilities of *A. ferrooxidans *that pertain to its use in industrial bioleaching, including its ability to oxidize both sulfur and iron, to resist low pH, and to live in environments with potentially toxic organic and inorganic chemicals. They also suggest that it has the ability to precipitate metals in anaerobic environments, which would be deleterious to copper bioleaching activity.

• Our analysis also prompts several unexpected predictions, some of which could potentially be useful in biomining such as the proposed connection between biofilm formation and central carbon metabolism and the presence of several predicted quorum sensing mechanisms. Indications of past phage infection and conjugation events suggest potentially fruitful approaches for the development of efficient methods for genetic manipulation of this microorganism.

• Metabolic models also indicate how this microorganism could play an important role as a producer of fixed carbon and nitrogen and as a recycler of metals in bioleaching operations as well as in natural environments.

## Methods

### Genome sequencing and assembly

The genome was sequenced and assembled using the whole genome shotgun method as previously described [135-137].

### Sequence annotation

Gene modeling was performed using CRTICA [138] and GLIMMER [139]. The lists of open reading frames (ORFs) generated by both strategies were merged using CRITICA start sites when models were identical. The translated ORFs were submitted to BLAST analysis against the UNIPROT database to evaluate overlaps and alternative start sites. The final list of coding sequences (CDSs) was translated, and these amino acid sequences were then used to query the following databases (August-December, 2007): National Center for Biotechnology Information (NCBI) nonredundant database, UniProt, TIGRFam, Pfam, PRIAM, KEGG, COG, and InterPro. Manual functional assignments were performed gene-by-gene, when needed. Comparative genome analyses were also performed using the Comprehensive Microbial Resource [140].

### Nucleotide sequence accession numbers

The sequence and annotation of the complete *A. ferrooxidans *strain ATCC 23270 genome is available at the Comprehensive Microbial Resource (CMR) (J. Craig Venter Institute, ) and in GenBank/EMBL/DDBJ accession number CP001219.

## Authors' contributions

JE and DH proposed the study; RB provided the DNA; JE, RD and HT were responsible for the genome sequencing and assembly; all authors contributed to the annotation; JV was responsible for the metabolic reconstruction with contributions from RQ, IP and DH; JV and DH drafted the manuscript; all authors read and approved the final manuscript.

## Supplementary Material

Additional file 1 **Gene lists for amino acid metabolism, cofactor biosynthesis and purine and pyrimidine metabolism**. A list of genes in the *A. ferrooxidans *ATCC 23270 genome is provided that are predicted to be involved in amino acid metabolism, cofactor biosynthesis and purine and pyrimidine metabolism. Click here for file

Additional file 2 **Phylogram of four different Rubisco enzymes in various bacteria**. Phylogenetic tree of forms I (CbbL1, CbbL2), II (CbbM), and IV (RLP, Rubisco-like protein) of the large subunit of ribulose-1,5-bisphosphate carboxylase/oxygenase (RubisCO) from various organisms. The multiple sequence alignments and trees were produced with ClustalW and visualized with MEGA. Bootstrap values indicated at the nodes are based on 1,000 trials. The RubisCOs from *A. ferrooxidans *are highlighted by blue boxes. Species names are as follows: *Archaeoglobus fulgidus*, *Bacillus subtilis*, *Chlorobium limicola*, *Chlorobium tepidum*, *Hydrogenovibrio marinus*, *Ralstonia eutropha*, *Rhodobacter capsulatus*, *Rhodobacter sphaeroides*, *Rhodopseudomonas palustris*, *Rhodospirilum rubrum*, *Synechococcus *sp. PCC6301, *Thiobacillus intermedius*, and *Thiobacillus neapolitanus*. Click here for file

Additional file 3 **Gene lists and predicted properties for several metabolic and cellular processes**. This data provides a list of genes and predicted properties for transposase families, hydrogenase systems, nutrient transport systems, osmotic balance, heavy metal resistance systems and toxic organic compounds extrusion systems deduced from the *A. ferrooxidans *ATCC 23270 genome sequence. Click here for file

Additional file 4 **Gene clusters predicted to be involved in nitrogen metabolism**. This data provides a schematic representation of some of the gene clusters and genes predicted to be involved in nitrogen fixation. Click here for file

Additional file 5 **Genes predicted to be involved type IV pilus formation**. This data provides a list of genes predicted to be involved type IV pilus formation. Click here for file
