# Supplementary figures and images for: Acidithiobacillus ferrooxidans metabolism: from genome sequence to industrial applications
Source: BMC Genomics. 2008 Dec 11;9:597. doi: 10.1186/1471-2164-9-597 (PMC2621215; doi:10.1186/1471-2164-9-597)

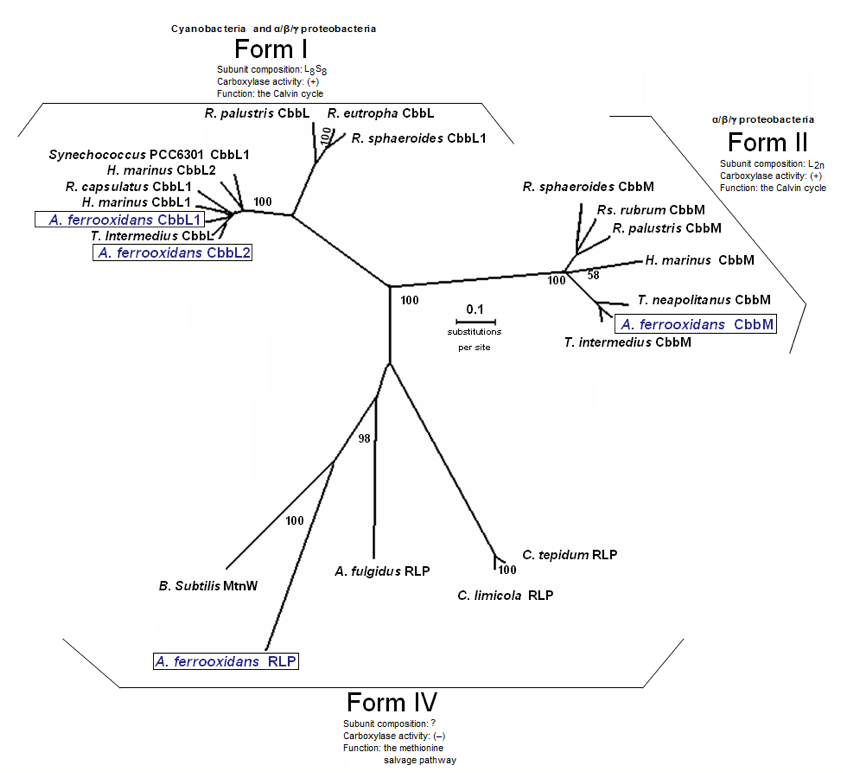

Supplement: Additional file 2 — Phylogram of four different Rubisco enzymes in various bacteria. Phylogenetic tree of forms I (CbbL1, CbbL2), II (CbbM), and IV (RLP, Rubisco-like protein) of the large subunit of ribulose-1,5-bisphosphate carboxylase/oxygenase (RubisCO) from various organisms. The multiple sequence alignments and trees were produced with ClustalW and visualized with MEGA. Bootstrap values indicated at the nodes are based on 1,000 trials. The RubisCOs from A. ferrooxidans are highlighted by blue boxes. Species names are as follows: Archaeoglobus fulgidus, Bacillus subtilis, Chlorobium limicola, Chlorobium tepidum, Hydrogenovibrio marinus, Ralstonia eutropha, Rhodobacter capsulatus, Rhodobacter sphaeroides, Rhodopseudomonas palustris, Rhodospirilum rubrum, Synechococcus sp. PCC6301, Thiobacillus intermedius, and Thiobacillus neapolitanus. [file 1471-2164-9-597-S2.png]

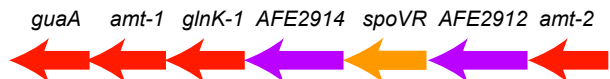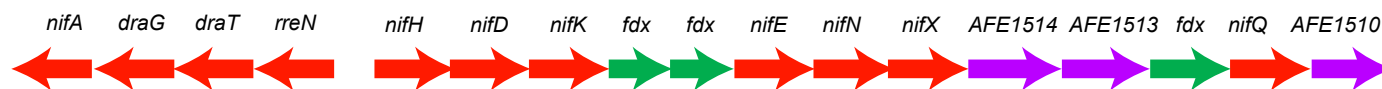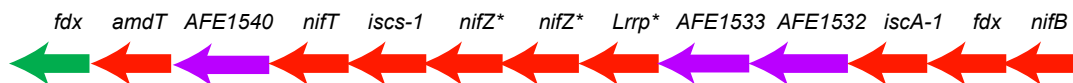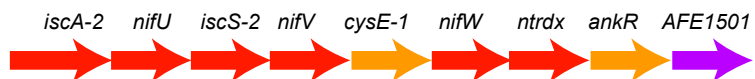

Nitrogen metabolism

Other functions

Hypothetical proteins

Ferredoxin

Supplement: Additional file 4 — Gene clusters predicted to be involved in nitrogen metabolism. This data provides a schematic representation of some of the gene clusters and genes predicted to be involved in nitrogen fixation. [file 1471-2164-9-597-S4.pdf]
