# Supplementary material for: Acidithiobacillus ferrooxidans metabolism: from genome sequence to industrial applications
Source: BMC Genomics. 2008 Dec 11;9:597. doi: 10.1186/1471-2164-9-597 (PMC2621215; doi:10.1186/1471-2164-9-597)
Supplement: Additional file 3 — Gene lists and predicted properties for several metabolic and cellular processes. This data provides a list of genes and predicted properties for transposase families, hydrogenase systems, nutrient transport systems, osmotic balance, heavy metal resistance systems and toxic organic compounds extrusion systems deduced from the A. ferrooxidans ATCC 23270 genome sequence. [file 1471-2164-9-597-S3.pdf]

Transposase families present in the *A. ferrooxidans* genome sequence.

Sequence group	Mahillon and Chandler family classification (129)	Size (bp)	Copy number	% Sequence nucleotide identity	Gene ID
ISAfe1	NF	NF	NF	NF	NF
ISAfe2	Ist2	1263	1	NF	AFE1330
ISAfe3	Is110	1227	8	100	AFE2972. AFE2709, AFE2432, AFE1265, AFE1142, AFE1110, AFE0550, AFE3263
ISAfe4 (orf1, 2 and 3)	Is3	2358	3	>96	AFE2939, AFE2468 AFE1709, AFE2938 AFE2469, AFE1708 AFE2470, AFE1706 AFE2937
ISAfe5 (orfA and B)	Is3	940	1	NF	AFE2393, AFE2394
ISAfe6	Is630	693	3	NF	AFE1655, AFE3105†, AFE3157†
ISAfe7 (orfA and orfB)	Is3	1241	1	NF	AFE1610, AFE1608
ISAfe8	Is200/Is605	1227	2	>99	AFE1215, AFE1058
ISAfe9 (transposase and transposition helper protein)	Is21	1485	1	NF	AFE3177, AFE3176
ISAfe10	Is110	1029	2	100	AFE1895, AFE3183

NF = Not found; †two degenerate copies with N-terminal similarity.

Predicted hydrogenase systems.

Genome ID	Gene name	Predicted function	Predicted location	Predicted TM* domains	Domains and motifs
**Group 4 hydrogenase**					
AFE2154	*hyfI*	hydrogenase-4, I subunit, [NiFe]-hydrogenase-3-type complex, small subunit/NADH:quinone oxidoreductase	cytoplasm	-	PF01058,PS00527,PIRSF002913,PIRSF500034,G3DSA:3.40.50.700,PTHR11995:SF3,PTHR11995
AFE2153	*hyfG*	hydrogenase-4, G subunit, [Ni-Fe] hydrogenase, Nickel-dependent hydrogenase, large subunit	cytoplasm	-	PTHR11993,PF00329,PF00346,G3DSA:1.10.645.10
AFE2152	*hyfF*	hydrogenase-4, F subunit, (NADH-ubiquinone oxidoreductase)	membrane	12	PF00361,PTHR22773:SF40
AFE2151	*hyfE*	hydrogenase-4, E subunit	membrane	7	
AFE2150	*hyfC*	hydrogenase-4, C subunit, (Respiratory-chain NADH dehydrogenase)	membrane	8	PTHR11432
AFE2149	*hyfB*	hydrogenase-4, B subunit, (NADH-ubiquinone oxidoreductase)	membrane	17	PR01434,PF00361,PTHR22773:SF13
**Group 3 hydrogenase**					
AFE0937	*hoxH*	[NiFe] hydrogenase, beta subunit, (4Fe-4S ferredoxin, iron-sulfur binding)	cytoplasm	-	PS00198
AFE0939	*hoxY*	[NiFe] hydrogenase, gamma subunit, (Cytochrome-c3 hydrogenase)	cytoplasm	-	PF00970,PF00175,PIRSF006816,PTHR11938:SF7,PR00406,G3DSA:3.40.50.80
AFE0938	*hoxU*	[NiFe] hydrogenase, delta subunit, (NADH ubiquinone oxidoreductase)	cytoplasm	-	PF01058,G3DSA:3.40.50.700
AFE0937	*hoxF*	[NiFe] hydrogenase, alpha subunit, (Nickel-dependent hydrogenase, large subunit)	cytoplasm	-	PF00374,G3DSA:1.10.645.10,PS00508
**Group 2 hydrogenase**					
AFE0701	*hupS*	hydrogenase-2, small subunit	cytoplasm	-	PF08425,PF01058,3DSA:4.10.480.10,G3DSA:3.40.50

		(Nickel-dependent hydrogenase, small subunit)			.700,PR00614
AFE0702	*hupL*	hydrogenase-2, large subunit (Nickel-dependent hydrogenase, large subunit)	cytoplasm	-	PF00374,G3DSA:1.10.645.10,PS00507
**Group 1 hydrogenase**					
AFE3286	*hynL*	[Ni-Fe] hydrogenase, large subunit	cytoplasm	-	PS00507,PS00508,PTHR11993,PF00374,G3DSA:1.10.645.10
AFE3285	*isp1*	4Fe-4S ferredoxin, iron-sulfur cluster-binding protein	cytoplasm	-	G3DSA:1.10.1060.10,PF00037,PF02754,PS00198
AFE3284	*isp2*	membrane protein	membrane	5	
AFE3283	*hynS*	[Ni-Fe] hydrogenase, small subunit	periplasm	-	G3DSA:3.40.50.700,G3DSA:4.10.480.10,PR00614,PF08425,PF01058,TIGR00391,TIGR01409

*TM = Transmembrane

Predicted nutrient transport systems

Inorganic compound transport systems		Nº of genes	Gene ID
**Sulfate**			
Sulfate permease/carbonic anhydrase			
	Sulfate permease	1	AFE0286
	Carbonic ahydrase	1	AFE0287
**Phosphate**			
Phosphate ABC transport			
	Permease protein	4	AFE1941, AFE1940, AFE1438, AFE1437
	Periplasmic phosphate binding protein	2	AFE1939, AFE1436
	ATP binding protein	1	AFE1439
**Iron**			
TonB dependent transport			
	ExbB	7	AFE3003, AFE2299, AFE2273, AFE2270, AFE1485, AFE0768, AFE0485
	ExbD	6	AFE3004, AFE2300, AFE2269, AFE1486, AFE0769, AFE0486
	TonB	7	AFE3002, AFE2304, AFE2301, AFE2275, AFE2268, AFE1487, AFE0770
	TonB dependent receptor	11	AFE2935, AFE1483, AFE1492, AFE2040, AFE2998, AFE2302, AFE2298, AFE2292, AFE2288, AFE0763, AFE3229
Fe/Mo ABC transport			
	Permease protein FecCD family	2	AFE1495, AFE1490
	ATP binding protein	1	AFE1491
	Periplasmic iron binding protein	2	AFE1494, AFE1489
	Periplasmic molybdate binding protein	1	AFE1493
Fe II FeoABC			

Ferrous iron transport protein A	1	AFE2523
Ferrous iron transport protein B	1	AFE2524
Ferrous iron transport protein C	1	AFE2525
MntH system		
MntH system	1	AFE0105
Porins		
FeoP	1	AFE2522
**Ammonium**		
Ammonium permeases		
Amt family	2	AFE2916, AFE2911
Amt-B family	1	AFE1922
**Organic compound transport systems**		
Amino acid transport		
Amino acid permease	5	AFE2659, AFE2457, AFE1782, AFE0719, AFE0489
Dipeptide ABC transport		
Permease protein	2	AFE2991, AFE2990
Periplasmic peptide binding protein	2	AFE2992, AFE2987
ATP binding protein	2	AFE2989, AFE2988
Carbohydrate transport		
PTS system	1	AFE3018-AFE3023
Carbohydrate selective porins	1	AFE2250
MFS sugar transporter	1	AFE2312
Sugar permease LacY family	1	AFE1971
Nucleotides and nucleosides		
cytosine/purines/uracil/thiamine/allantoin permease family protein	1	AFE0771
Phosphonate		
Phosphate ABC transport		

	ATP binding protein	2	AFE2285, AFE2283, AFE2282

Predicted components involved in osmotic balance

**Predicted K^+, Na^+, Mg^{2+}, Co^{2+}, Cl^-, and H^+ transport**	**N° of genes**	**Gene ID**
Na^+/H^+ antiporter	4	AFE2454, AFE2205, AFE2245, AFE0791
Na^+/Ca^{2+} exchanguer	1	AFE0587
Mg^{2+} translocating P-type ATPase	1	AFE2327
Mg^{2+} and Co^{2+} transport protein	4	AFE0599, AFE3221, AFE3219, AFE3218
H^+ efflux P-type ATPase	2	AFE3086, AFE0818
Voltage-gated potassium K^+ channel	2	AFE3093, AFE2922
K^+ uptake protein	1	AFE1753
K^+ efflux system protein	1	AFE1968
Kdp ATPase system protein A	3	AFE2477, AFE2475, AFE2232
Kdp ATPase system protein B	2	AFE2474, AFE2233
Kdp ATPase system protein C	2	AFE2473
Kdp ATPase system protein F	2	AFE2476, AFE2231
Voltage gated Cl^- channel	3	AFE0815, AFE0814, AFE3129
Mechanosensitive ion channel family protein	1	AFE1784
Large conductance mechanosensitive channel protein	1	AFE3260
**Undefined cation transport**		
Cation ABC transporter, permease protein	2	AFE0605, AFE0604
Cation ABC transporter, ATP-binding protein	1	AFE0603
Cation ABC transporter, periplasmic binding	1	AFE0602

protein		
Cation diffusion facilitator family transporter	3	AFE2420, AFE1496, AFE1430
Cation efflux family protein	1	AFE0465
Cation efflux permease	1	AFE2424
Cation efflux system protein	1	AFE2782
Metal ion transporter	2	AFE0920, AFE0105

Predicted heavy metal resistance systems

Heavy metal resistance and efflux		Gene ID
	**Ars system**	
	Arsenate reductase (*arsC*)	AFE2860
	Arsenate represor (*arsR*)	AFE2859
	Arsenate efflux pump (*arsB*)	AFE2858
	Unknown function (*arsH*)	AFE2857
	**Mer system**	
	Mercury co-repressor (*merD*)	AFE2483
	Hg^{2+} reductase (*merA*)	AFE2481
	Hg^{2+} uptake protein (*merC*)	AFE2480
**Heavy metal efflux**		
	Copper translocating P-type ATPase (3)	AFE2779, AFE2439, AFE2021
	Copper resistance protein CopC	AFE2650
	Copper resistance protein CopD	AFE2651
	Heavy metal efflux outer membrane protein (2)	AFE1949, AFE2434
	Heavy metal efflux pump CzcA family (2)	AFE2431, AFE1947
	Heavy metal efflux transporter MFP subunit (2)	AFE2433. AFE1948

Cation-transporting P-type ATPase (2)	AFE2944, AFE1988

Predicted toxic organic compounds extrusion systems

Drug efflux	N° of genes	Gene ID
Toluene transport system		
TolB protein	1	AFE0067
TolA protein	1	AFE0066
TolR protein	1	AFE0065
TolQ protein	1	AFE0064
Toluene tolerance protein	2	AFE1830, AFE0161
Toluene ABC transporter ATP binding protein	2	AFE0163, AFE0158
Toluene transport system Ttg2E protein	1	AFE0162
Toluene tolerance ABC-system periplasmic component	1	AFE0157
Toluene tolerance protein Ttg2B, putative	1	AFE0156
Fosmidomycin resistance protein, putative	1	AFE1977
AmpG permease protein, putative	1	AFE1961
Outer membrane protein, OMPP1-FadL-TodX family	1	AFE2542
Drug resistance transporter, EmrB-QacA family	7	AFE2184, AFE2091, AFE1797, AFE2124, AFE0864, AFE0040, AFE0161
RND drug efflux system, outer membrane lipoprotein	2	AFE0863, AFE0039
Drug resistance secretion protein	1	AFE0038

ABC multidrug efflux system		
ABC transporter, permease protein	3	AFE2864, AFE2863, AFE1603
ABC transporter, ATP-binding protein	3	AFE2862, AFE1606, AFE1605
Secretion protein, HlyD family	3	AFE2166, AFE2861, AFE1607
Dicarboxylate transport		
C4-dicarboxylate transporter-malic acid transport protein	3	AFE1992, AFE1847, AFE0041
