# Supplementary material for: Acidithiobacillus ferrooxidans metabolism: from genome sequence to industrial applications
Source: BMC Genomics. 2008 Dec 11;9:597. doi: 10.1186/1471-2164-9-597 (PMC2621215; doi:10.1186/1471-2164-9-597)
Supplement: Additional file 5 — Genes predicted to be involved type IV pilus formation. This data provides a list of genes predicted to be involved type IV pilus formation. [file 1471-2164-9-597-S5.pdf]

Genes involved in type IV pilus biosynthesis

AFE2710	-	Pilin
AFE2708	*cpaA*	Flp pilus assembly protein protease CpaA
AFE2707	*tadG*	Pilus assembly protein
AFE2705	*cpaB*	Flp pilus assembly protein
AFE2704	*cpaC*	Flp pilus assembly protein secretin
AFE2703	*cpaE*	Pilus assembly protein CpaE
AFE2702	*tadA*	Tight adherence protein TadA
AFE2701	*tadB*	Tight adherence protein TadB
AFE2700	*tadC*	Tight adherence protein TadC
AFE2699	*tadD*	Flp pilus assembly protein TadD
AFE0973	-	Type IV pilin biogenesis peptidase
AFE0972	*pilC*	Type IV pilus biogenesis protein PilC
AFE0971	-	Type IV pilus biogenesis protein
AFE0970	-	Type IV pilin
AFE0969	-	Type IV pilin
AFE0968	-	Hypothetical protein
AFE0967		Type IV pilin biogenesis protein
AFE0739	*pilM*	Type IV pilus assembly protein PilM
AFE0738	*pilN*	Type IV pilus biogenesis protein PilN
AFE0737	*pilO*	Fimbrial assembly protein PilO
AFE0736	*pilP*	Type 4 fimbrial biogenesis protein PilP
AFE0735	*pilQ*	Fimbrial assembly protein PilQ
AFE0416	-	Pilin
AFE0186	*pilD*	Type IV pilus prepilin peptidase PilD
AFE0185	*pilR*	Type IV fimbriae expression regulatory protein PilR
AFE0184	*pilS*	Sensor protein PilS
AFE0183	-	Type IV pilus assembly protein
AFE0007	-	Outer membrane usher protein
AFE0006	-	Pilus chaperone protein
